# Kidney Care for All: Addressing the Gaps in an Imperfect World—A Global Perspective on Improving Access to Kidney Care in Low-Resource Settings

**DOI:** 10.34067/KID.0000000000000128

**Published:** 2023-04-17

**Authors:** Priya Pais, Arpana Iyengar

**Affiliations:** Department of Pediatric Nephrology, St John's Medical College, St John's National Academy of Health Sciences, Bengaluru, India

**Keywords:** chronic kidney disease, access, affordability, awareness, CKD care, dialysis, equity, kidney replacement therapy, low- and low-middle–income countries (LLMIC), pediatric CKD, solutions

A 50-year-old man in a low-income country presented to a private sector referral hospital with edema and symptomatic uremia and was diagnosed with kidney failure. His hypertension was detected 10 years ago, but he did not pursue evaluation or treatment. Urgent hemodialysis (HD) was initiated, and the need for maintenance HD or peritoneal dialysis (PD) was discussed. The patient farmed a piece of land in a rural area, the sole earner for his family, and did not have any private health care insurance. Although eligible for government-funded public sector dialysis, the nearest center was 200 km away and offered HD but did not have a PD program. He was unable to afford the out-of-pocket costs of HD or PD in the private sector nephrology center that initiated his treatment.

This scenario poses questions that involve challenges in access to CKD care. Why did this man's kidney disease not get diagnosed earlier? What treatment options are available to him? What is his outcome likely to be? Realistically speaking, the patient will struggle to initiate maintenance dialysis, face unsurmountable difficulties associated with access to care, face catastrophic health care expenditure (CHE), drop out of dialysis, and die.^1,2^

This common scenario highlights the crucial importance of the World Kidney Day theme, Kidney Health for All, to improve patient outcomes. The authors conducted a panel discussion with leaders in nephrology and with experience of conditions in low- and low-middle–income countries (LLMICs), to discuss the issue of access to CKD care, as a Women in Nephrology (India) activity. The highlights of this discussion are summarized here.

## Practicing Nephrology in Low-Resource Settings—Trials and Triumphs

Despite the higher burden of CKD in LLMICs, there is a significant shortage of nephrology services, especially the capacity to provide KRT.^3^ CKD care is performed under difficult circumstances with higher patient numbers, more severe morbidity, and greater mortality than in high-income countries. Working within twin constraints of patient poverty and limited resources, physicians are forced to accept suboptimal therapy (*e.g.*, twice weekly dialysis) which allows more patients to survive but denies them a chance to thrive. When there is no universal health care coverage (UHC) for KRT, there is an unfair disparity in patient outcomes between the public sector with its limited resources and the highly resourced private sector, which serves the few who can afford care or have insurance.

Yet, many in nephrology persist and thrive in these conditions—compensating for the challenges with the satisfaction of providing care to those who are most in need. The goals of treatment remain the same, to achieve the best possible patient outcomes under their circumstances. The availability of generic medications in many countries makes medical therapy more affordable than in high-income countries. Working with patients who have low health awareness requires nephrologists to play an active role in decision-making with the family. Although this close involvement with patients can become a source of moral distress, nephrologists gain an in-depth perspective of the real-world challenges of CKD care. They also become familiar with governmental and nongovernmental agencies involved in funding treatment in their quest to find financial support for their patients and families. This familiarity with all key stakeholders gives nephrologists a unique advantage for advocacy.

## Challenges in Access to CKD Care

**Figure 1:**
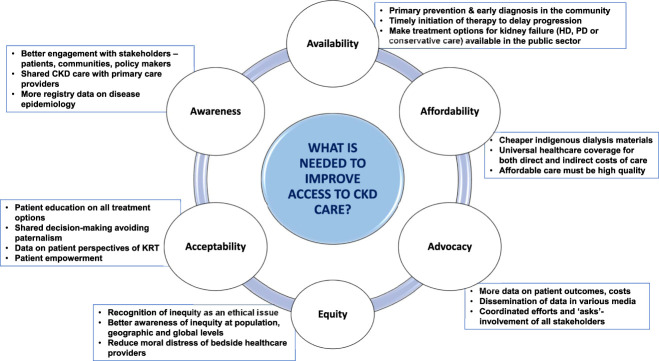
Unmet needs of access to CKD care in low- and low-middle–income countries.

Having access to CKD care implies health care services be not just available, but also in adequate supply to meet demand, effective to accomplish goals of treatment and relevant to ensure good patient outcomes.^4^ In LLMICs, access to care is impeded by several challenges described below (Figure 1).

### The Lack of Awareness of CKD

The lack of awareness of CKD as an important and life-threatening noncommunicable disease is prevalent in the population, its leaders, policy makers, and primary health care providers. Even after CKD diagnosis, the awareness of their own disease in patients with CKD is low.^5^ Delayed diagnosis denies patients the opportunity to retard progression. Therefore, there is a lack of primary and secondary prevention programs in the community and health care system. This shifts the burden of care of advanced, untreated patients with CKD onto nephrologists who must not only reveal a CKD diagnosis but also simultaneously assist in clinical decision-making about KRT.

There are no national CKD or kidney failure registries in most LLMICs, including India. This is due to the shortage of funds to run such large registries, absent UHC for KRT, and therefore no centralized electronic health record or administrative health care database.^6^ Although nephrologists practicing in these settings are familiar with problems associated with delayed diagnosis of CKD and the high rates of mortality among dialysis patients, there are no hard data to present to policy makers to change the *status quo*.

### Limited Availability of KRT

Dialysis and transplant centers are concentrated in urban areas, forcing patients in rural or remote areas to travel long distances.^7^ Owing to low public budgetary health care expenditure, free public sector dialysis facilities are available for only a fraction of the population with kidney failure, resulting in rationed care.^8^ In addition, getting a dialysis HD bed does not assure access to the necessary consumables, manpower, or quality of care. PD, which would be ideal for a rural/remote patient population, is not available or is underutilized in many LLMICs. Conservative care programs (kidney failure management without dialysis) for those unwilling/unable to undergo dialysis are not routinely available. Availability of kidney transplantation in most LLMICs is very limited or nonexistent.

### Lack of Affordability

Currently, KRT is unrealistically expensive, especially for patients in low-resource settings, resulting in CHE for patients and their families. With costly consumables and solutions produced by large pharmaceutical companies, dialysis is not sustainable for the majority who pay out of pocket. Besides the direct costs, many patients living in remote areas must pay for travel for themselves and an accompanying family member/carer to receive care that is concentrated in cities. There are no social support systems to cover the indirect costs associated with kidney care. Thus, even with free KRT, patients are burdened with CHE.^7^ Health care sectors of LLMICs have not yet invested in making dialysis cheaper. Although good quality generic medications may be available at lower cost than branded drugs, they are still expensive when patients must pay out of pocket to purchase them when they are not provided free by the government. Research and innovation are not pursued because of a lack of support and funding. Faced with this stark reality, nephrologists must consider that dialysis, although lifesaving, may not be an ideal treatment for those who are unable to afford it without the risk of impoverishment.

### Inequity

Inequities in health care occur when there are unfair, avoidable, and remediable differences between groups of people on the basis of socioeconomic, demographic, or geographic factors.^9^ In countries where public sector KRT must be rationed due to limited availability, the burden of choosing who lives or dies results in moral distress for clinicians.^10^ There is a need for ethical priority setting to ensure a fair and equitable access to care. In regions without UHC, the ability to pay determines survival. In these circumstances, physicians are faced with the ethical dilemma of advising the best treatment for the patient versus considering the best interest of the family.

## Special Challenges in Pediatric Nephrology

Children develop CKD from congenital anomalies of the kidney and urinary tract, not from traditional adult risk factors. Without coordinated perinatal care, serious anomalies do not receive the required early intervention. Pediatric CKD is usually from nonglomerular disease, and the subtle symptoms—nocturia, poor growth, and anemia—are not recognized or evaluated until advanced CKD has occurred. The care of pediatric CKD is specialized requiring attention to small body size, nutritional needs, growth, and cognitive development. Unfortunately, pediatric nephrology centers are sparse in LLMICs, and outcomes of children with kidney failure are poor. In low-resource settings, caring for children with lifelong disease is low priority for policy makers, society, and even caregivers who must balance needs of the one against all others in the family. The ethical dilemmas in pediatric nephrology are unique because children depend on their caregivers and physicians for decision-making, and the best interest is viewed differently and is often a source of conflict.^11^

## Real-World Solutions to Improve Access to CKD Care

The summary of this free-spirited sincere discussion on the limited access to CKD care in low-resource settings highlights real-world challenges. Although long-term and large-scale solutions to improve access to CKD care have been published, actual actionable steps are needed. Nephrologists are required to step out of their comfort zones in tertiary centers and build partnerships with various stakeholders in the community. Table 1 outlines practical suggestions achievable in low-resource settings to improve accessibility and patient outcomes.

**Table 1:** Practical solutions to improving access to CKD care in low- and low-middle–income countries

Area for Improvement	Nephrology Partnerships	Actionable Steps
Awareness	Primary health care Tertiary health care Local leaders Community Funding organizations	1. Innovate with community health workers to drive primary and secondary prevention a. Screening for risk factors b. Checklist-based treatment of risk factors 2. Tertiary health care: Liaise with other specialists to improve screening in patients with comorbidities and risk factors for CKD. This will improve early diagnosis and facilitate therapy to retard CKD progression. 3. Interact with local leadership and health care units to drive awareness campaigns 4. Conduct education campaigns to educate the community regarding CKD risk factors and need for screening and early diagnosis in those at risk. 5. Petition to recognize CKD as an important NCD to achieve sustainable development goals, specifically SDG 3 (good health and well-being)
Availability	Public+private sector	1. Petition government policy makers to reimburse private sector kidney care, taking advantage of infrastructure and quality of care. 2. Increase patients' access to nephrologists by encouraging task shifting (delegation of aspects of care) to less specialized personnel, *e.g.*, shifting the task of screening for hypertension to community health workers 3. Include allied personnel in the nephrology workforce to provide more holistic care with less focus on dialysis as procedure, *e.g.*, nurses, dieticians, social workers, psychologists 4. Insist on availability of affordable drugs required for prevention/slowing progression of CKD 5. Initiate conservative care programs in all centers to focus on patient-centered outcomes
Affordability Innovation	Research organizations Engineers and scientists	1. Leverage grant funds from nonpharmaceutical sources to study cheaper treatments 2. Use ubiquitous mobile technology to enhance patient–provider interactions without travel 3. Collaborate with innovators to research effective, inexpensive technologies for dialysis 4. Accept locally manufactured dialysis hardware and consumables to reduce import duties 5. Perform implementation research to determine which innovations might achieve treatment goals
Affordability Advocacy for UHC Ensuring equity	Policy makers Patients Patient support groups	1. Petition local and national health care policy makers to provide financial coverage of essential kidney care by: a. Generating data from disease/patient registries b. Publishing in national and international media c. Discussion in public forums and using written petitions 2. Involve all stakeholders in priority setting to discuss health care coverage under the following pillars: a. Which disease, stage, and treatment? b. Which target population? c. How to prevent CHE?
Improving the quality of care	Primary care physicians Patients, caregivers Palliative care, social worker Psychologist	1. Enhance liaising with primary care physicians to improve awareness among patients of their CKD diagnosis and importance of treatment adherence 2. Improve shared decision-making between physicians and patients/caregivers. Identify what outcomes matter most 3. Create better conservative care frameworks for those unable or unwilling to undergo dialysis, maintaining 4. Focus more on QoL, self-reliance, resilience
Pediatric kidney care Awareness Advocacy Equity	Community workers in maternal child health/pediatricians Policy makers/adult specialist fraternity Community, caregivers	1. Extend prenatal care into follow-up throughout childhood. 2. Highlight the simple methods of identifying kidney disease in children (growth, BP measurement, rickets) 3. Interact with pediatricians and lay community to send message that kidney disease is treatable 4. Provide key reasons to refer and ensure easy referral systems 1. Advocate for UHC for pediatric CKD as a rare disease 2. Avoid the utilitarian approach to valuing the life of a child, instead giving children a fair innings at a chance for medical well-being 3. Ease caregiver burden by supporting families mentally, financially, and socially.
Patient empowerment	Patients, caregivers, and their communities	1. Communicate meaningfully not just the facts of CKD diagnosis and management but also explain its lifestyle implications and the treatment options available. 2. Encourage independent decision-making by women and older patients who are frequently side-lined when treatment decisions must be taken. 3. Liaise with allied health team members such as social workers to encourage formation of patient support groups for peer interaction and sharing. 4. Advise patients, families, and patient support groups to advocate with policy makers for their key needs. 5. Advocate for patient support groups to be included as key stakeholders during policymaking

LLMIC, low- and low-middle–income countries; NCD, noncommunicable disease; SDG, sustainable development goals; UHC, universal healthcare coverage; CHE, catastrophic healthcare expenditure; QoL quality of life.

## Disclosures

A. Iyengar reports the following—advisory or leadership role: Member of the Editorial board for Frontiers in Pediatrics and Peritoneal Dialysis International and Chair of the Clinical Research Program of the International Society of Nephrology 2021–2023 and other interests or relationships: Member of the IPNA, IPTA, ISN, ISRNM, TTS, and WIN. P. Pais reports the following—advisory or leadership role: I serve on the following committees of international societies. These positions are not paid; IPTA—Outreach Committee; IPNA—Sister Renal Center Committee.
